# The Role of the Mechanical, Structural, and Thermal Properties of Poly(l-lactide-*co*-glycolide-*co*-trimethylene carbonate) in the Development of Rods with Aripiprazole

**DOI:** 10.3390/polym13203556

**Published:** 2021-10-15

**Authors:** Artur Turek, Jakub Rech, Aleksandra Borecka, Justyna Wilińska, Magdalena Kobielarz, Henryk Janeczek, Janusz Kasperczyk

**Affiliations:** 1 Chair and Department of Biopharmacy, Faculty of Pharmaceutical Sciences in Sosnowiec, Medical University of Silesia, Katowice, Jedności 8, 41-200 Sosnowiec, Poland; justyna.wilinska@med.sum.edu.pl (J.W.); janusz.kasperczyk@sum.edu.pl (J.K.); 2 Department of Biotechnology and Genetic Engineering, Faculty of Pharmaceutical Sciences in Sosnowiec, Medical University of Silesia, Katowice, Jedności 8, 41-200 Sosnowiec, Poland; jrech@sum.edu.pl; 3 Centre of Polymer and Carbon Materials, Polish Academy of Sciences, M. Curie-Skłodowskiej 34, 41-819 Zabrze, Poland; aborecka@cmpw-pan.edu.pl (A.B.); hjaneczek@cmpw-pan.edu.pl (H.J.); 4 Department of Mechanics, Materials and Biomedical Engineering, Faculty of Mechanical Engineering, Wrocław University of Science and Technology, Wybrzeże Wyspiańskiego 27, 50-370 Wroclaw, Poland; magdalena.kobielarz@pwr.edu.pl

**Keywords:** aripiprazole, poly(l-lactide-*co*-glycolide-*co*-trimethylene carbonate), drug delivery system, polymer degradation, mechanical properties, compression and tensile tests, shape memory

## Abstract

In this work, we aimed to determine the role of the mechanical, structural, and thermal properties of poly(l-lactide-*co*-glycolide-*co*-trimethylene carbonate) (P(l-LA:GA:TMC)) with shape memory in the formulation of implantable and biodegradable rods with aripiprazole (ARP). Hot melt extrusion (HME) and electron beam (EB) irradiation were applied in the formulation process of blank rods and rods with ARP. Rod degradation was carried out in a PBS solution. HPLC; NMR; DSC; compression and tensile tests; molecular weight (*M_n_*); water uptake (*WU*); and weight loss (*WL*) analyses; and SEM were used in this study. HME and EB irradiation did not influence the structure of ARP. The mechanical tests indicated that the rods may be safely implanted using a pre-filled syringe. During degradation, no unfavorable changes in terpolymer content were observed. A decrease in the glass transition temperature and the *M_n_*, and an increase in the *WU* and the *WL* were revealed. The loading of ARP and EB irradiation induced earlier pore formation and more intense *WU* and *WL* changes. ARP was released in a tri-phasic model with the lag phase; therefore, the proposed formulation may be administered as a delayed-release system. EB irradiation was found to accelerate ARP release.

## 1. Introduction

Aripiprazole (ARP) is an atypical antipsychotic with a unique receptor-binding profile. Partial agonist activity at D_2_ and 5-HT_1A_ receptors and potent antagonism at 5-HT_2A_ receptors characterize ARP pharmacodynamics, which determine lower extrapyramidal side-effects and the overall effectiveness of pharmacotherapy of schizophrenics [1]. However, in mental disorders, the formulation and administration are as important as the drug substance itself. Therefore, parenteral formulations with a prolonged release are preferred and have been proposed in the last decade [2]. One popular line of research is the use of biodegradable polymers. ARP in poly(caprolactone) nanoparticles [3], poly(lactide-*co*-glycolide) (PLGA) microparticles [4,5], polylactide microparticles [6], and PLGA implants in situ [7] have been proposed. However, small formulations such as microparticles are administered as aqueous suspensions, which may cause pain and cannot be removed if side effects appear. Moreover, problems such as an unfavorable burst effect, microparticle aggregation, and low loading efficiency may be observed. Therefore, in this study implantable and biodegradable rods based on poly(l-lactide-*co*-glycolide-*co*-trimethylene carbonate) (P(l-LA:GA:TMC)) with ARP (P(l-LA:GA:TMC) rod-ARP) administered with a pre-filled syringe were developed as an alternative to the known solutions. Previously, it was noted that P(l-LA:GA:TMC), which has similar structural properties, was able to move from a temporarily fixed shape back to its original permanent shape upon exposure to a thermal stimulus [8], which can be a significant advantage in administration. This feature may reduce invasiveness because of the smaller diameter and greater length of the formulation before implantation, as well as its greater diameter and smaller length after implantation, as has been discussed previously [8,9].

Hot melt extrusion (HME) is one of the methods of rod formulation used for the processing of thermoplastic materials [9,10,11,12]. The flow temperature of polymers was primarily determined empirically. However, it should not exceed the melting temperature (*T_m_*) of the drug substance [9,10]. In turn, electron beam (EB) irradiation is the sterilization method that is most often used for biomaterials and implantable drug formulations [13,14]. It should be noted that EB irradiation may influence the structural properties of polymers and can be used to tailor their properties. However, it cannot change the structure of the drug substance [13,15].

The design of rods based on P(l-LA:GA:TMC) rod-ARP requires (i) the selection of an appropriate formulation method, (ii) the study of the mechanical properties of the raw polymer carrier to determine the best method of administration, (iii) the study of the influence of the formulation method on the drug substance, and (iv) the influence of degradation on the structural properties of the polymer carrier and drug release.

It should be pointed out that rods may be administered subcutaneously or intramuscularly by implantation with a pre-filled syringe, which subjects the rods to mechanical stress. Therefore, the compression and tensile testing of polymer carriers provides essential data on their ductile or brittle modes [16]. It is therefore crucial to define the risk of uncontrolled formulation destruction or decomposition and uncontrolled drug release. One can make the hypothesis that for rods, the ductile mode is preferred to the brittle mode due to the lower risk of polymer damage (breaking, fracturing, or microcrack propagation) and material decomposition.

This study aimed to determine the role of the mechanical, structural, and thermal properties of P(l-LA:GA:TMC) in the formulation of an implantable and biodegradable rod with ARP.

## 2. Materials and Methods

### 2.1. Terpolymer

P(l-LA:GA:TMC), with tailored features (Table 1), was synthesized in bulk with the use of a low-toxicity initiator, Zr(Acac)_4_ (Sigma-Aldrich, Poznań, Poland) [10].

### 2.2. Rod Formulation

Blank rods (1 mm × 10 mm; *n* = 20) based on P(l-LA:GA:TMC) (P(l-LA:GA:TMC) rods) and rods with 10% *w*/*w* of ARP (Zhejiang Huahai Pharmaceutical Co., Ltd., Linhai City, China) (P(l-LA:GA:TMC) rods-ARP) (1 mm × 10 mm; *n* = 20) were formulated by HME in a co-rotating twin-screw extruder (Thermo Scientific, Haake MiniLab II, Karlsruhe, Germany).

Before the process, raw terpolymer was air-dried and subjected to grinding at a temperature of −196 °C in a cryogenic mill (6870 SPEX, Thermo Fisher Scientific, Ottawa, ON, Canada). Then, ARP was introduced to the milled terpolymer. The mixture was vortexed and subsequently placed in a vacuum oven with a temperature of 23 °C and a pressure of 80 mbar for 14 days, then fed to an extruder cylinder heated to 105 °C. This process was carried out in a co-rotating twin screw extruder (Thermo Scientific, Haake MiniLab II, Karlsruhe, Germany) using a plasticizing screw rotational speed of 20 rpm. The molten mixture was extruded through a 0.7 mm diameter die and chilled on a roll. Then, rods 1 mm in diameter and 10 mm in length were formulated. P(l-LA:GA:TMC) rods were formulated according to the same procedure without ARP.

The EB irradiation of the rods was performed using an EB accelerator (10 MeV, 360 mA, 25 kGy) (The Institute of Nuclear Chemistry and Technology, Warsaw, Poland; Certificate no. 625/2017/E).

### 2.3. Rod Degradation

The rods were placed in a solution of phosphate buffered saline (PBS) (pH 7.4) (Sigma-Aldrich, Poznań, Poland) and incubated under constant conditions at a temperature of 37 °C with shaking at 240 rpm.

### 2.4. NMR Study

Proton nuclear magnetic resonance (^1^H NMR) spectroscopy was used in the study of (i) the ARP structure, (ii) the efficiency of the loading of ARP into P(l-LA:GA:TMC), and (iii) the P(l-LA:GA:TMC) composition. In each case, the ^1^H NMR spectra were recorded at 600 MHz with a Bruker Avance II Ultrashield Plus spectrometer (Karlsruhe, Germany) operating at 600 MHz using a 5 mm sample tube, 32 scans, an 11 μs pulse width, and a 2.65 s acquisition time.

**Figure 1:**
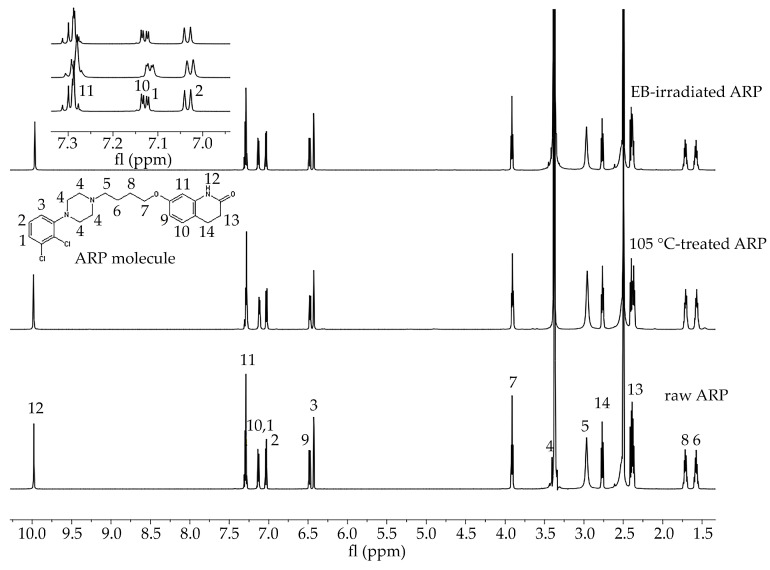
^1^H NMR spectra of raw ARP, 105 °C-treated ARP, and EB-irradiated ARP (DMSO-d6, 600 MHz). Signals observed in the spectra in the range 10.0 to 1.5 ppm were numbered from 1 to 14 and assigned to the appropriate hydrogen atom-containing molecular group present in the ARP structure. Signal no. 12: 9.98 (s, 1H, −NH), 11: 7.43–7.25 (m, 2H, −ArH), 10, 1: 7.20–7.09 (d, 1H, −ArH), 2: 7.03 (d, J = 8.3, 1H, −ArH), 9: 6.48 (dd, J = 8.2, 2.5, 1H, −ArH), 3: 6.43 (d, J = 2.4, 1H, −ArH), 7: 3.96–3.88 (m, 2H, CO−CH_2_CH_2_–), 4: 3.52–3.39 (m, 1H, CH_2_ of piperazine), 5: 3.06 (d, J = 115.8, 4H,−CH_2_ of piperazine), 14: 2.67 (m, 4H, −CH_2_ of piperazine), 13: 2.43–2.34 (m, 2H, −CH_2_ of piperazine), 8: 1.77–1.65 (m, 2H, -CH_2_), 6: 1.63–1.51 (m, 2H, −CH_2_). Signals between 7.3 and 7.0 ppm have been enlarged to offer better visibility.

In the study of the ARP structure, samples of raw ARP, 105 °C-treated ARP (3 min, reflecting HME conditions), and EB-irradiated ARP were analyzed. The signals observed in the ^1^H NMR spectra were assigned to the appropriate hydrogen atom-containing groups present in the ARP structure (Figure 1).

The efficiency of the loading of ARP into the terpolymer rods was calculated based on the calibration curve obtained using ^1^H NMR spectroscopy. Six concentrations of ARP in DMSO-d6 (1 μg/mL, 10 μg/mL, 50 μg/mL, 100 μg/mL, 1 mg/mL, and 5 mg/mL) were prepared and analyzed. The signals (1–14) found for the spectra shown in Figure 1 were assigned to the hydrogen-containing molecular groups present in the ARP structure. The integral value of the NH group signal (signal no. 12 in Figure 1) was used to calculate the loading efficiency of ARP. The calibration curve was prepared based on the ratio of the integral values of the signals of the NH group and DMSO-d6 (NH/DMS0-d6, *y*-axis), depending on the concentration of ARP in mg/mL. The efficiency of the ARP loading was calculated using Equation (1):

Loading efficiency (%) = (amount of ARP in rods)/(amount of ARP introduced into terpolymer) × 100(1)

For the composition study, the signals observed in the ^1^H NMR spectra were assigned to the appropriate sequences in the polymer chain. The molar percentages of the monomer units of lactide (*F_LL_*), glycolide (*F_GG_*), and carbonate (*F_TMC_*) were calculated according to a previously described procedure [17].

### 2.5. DSC Study

Differential scanning calorimetry (DSC) was used in the study of (i) the thermal properties of ARP, (ii) raw P(l-LA:GA:TMC), and (iii) rods. The measurements were performed using a DSC Q2000 apparatus (TA Instruments, New Castle, DE, USA) calibrated with high-purity indium in a nitrogen atmosphere (with a flow rate of 50 mL/min).

The samples were heated at a rate of 20 °C/min. During the first run, the samples were heated to 200 °C and then the melted samples were rapidly cooled to −20 °C. During the second run, the samples were heated within the range of −20 to 200 °C.

For the ARP samples, the values of the *T_m_* and the crystallization temperature (*T_c_*) of ARP were determined, whereas for the raw P(l-LA:GA:TMC) and rods the values of the *T_m_* and the glass transition temperature (*T_g_*), which was designated as the midpoint in the heat capacity change in the amorphous sample from the second heating run, were determined.

### 2.6. Mechanical Study

The mechanical properties of raw P(l-LA:GA:TMC) were determined in the compression and tensile tests according to the standards PN-EN ISO 604:2006 and EN ISO 527-1:2012, respectively. Both strength tests were carried out on a testing machine (858 MiniBionix, MTS Systems Headquarters, Minneapolis, MN, USA) with a constant speed of 1 mm/min.

The cylindrical specimens used for the compression tests (with dimensions 8 × 5 mm) were loaded axially. For the tensile test, dumbbell-shaped specimens (type 5A with dimensions 20 × 4 × 2 mm) were placed onto the testing machine using hydraulic-type grips at a constant clamping pressure of 3 bar.

The stress–strain characteristics were determined for each specimen, and the following mechanical parameters were calculated: compressive and tensile Young’s modulus, yield stress and strain (σ_y_, ε_y_), and breaking (identical to maximum) stress and strain (σ_b_, ε_b_).

### 2.7. Molecular Weight and Molecular Weight Distribution Study

The molecular weight (*M_n_*) and molecular weight distribution (*D*) of the rod samples were determined by gel permeation chromatography (GPC) using a Viscotek Rlmax chromatograph (Malvern Panalytical Ltd., Malvern, Worcestershire, UK) with two Viscotek 3580 columns and a Shodex SE 61 detector. The process was performed at a flow rate of 1 mL/min using chloroform as a solvent. The *M_n_* value was calibrated with polystyrene standards.

### 2.8. Water Uptake and Weight Loss Study

Changes in the water uptake (*WU*) and weight loss (*WL*) of the rods were determined using Equations (2) and (3), respectively:

*WU* (%) = (wet mass − dry mass)/(dry mass) × 100 (2)

*WL* (%) = (initial mass − dry mass)/(dry mass) × 100 (3)

### 2.9. Morphology Study

The rod morphology was characterized using a scanning electron microscope (SEM) (Quanta 250 FEG/FEI, Thermo Fisher Scientific, Waltham, MA, USA) operating with an acceleration voltage of 5 kV under low-vacuum conditions (80 Pa) from secondary electrons collected by a large field detector. The samples were mounted on microscope stubs with the use of double-sided adhesive carbon tape.

### 2.10. ARP Release

The ARP concentration was determined by high-performance liquid chromatography (HPLC) using an Elite LaChrom HPLC system (VWR Hitachi, Merck, Warsaw, Poland) with a UV–VIS detector (Diode Array Detector L-2355, VWR Hitachi, Merck, Warsaw, Poland) set at 240 nm with a column 5 µm in diameter (LiChrospher RP-18 250-4, Sigma-Aldrich, Poznań, Poland). The mobile phase was methanol (Sigma-Aldrich, Poznań, Poland) and ammonium acetate (Sigma-Aldrich, Poznań, Poland) (90:10, flow rate 1 mL/min).

## 3. Results and Discussion

In this study, HME and EB irradiation were applied in the formulation process of P(l-LA:GA:TMC) rods and P(l-LA:GA:TMC) rods-ARP. The results of the NMR and DSC studies showed that pointed methods are appropriate for rod formulation. Figure 1 presents an ^1^H NMR spectrum of raw ARP with signals in the range of 10.0 to 1.5 ppm. The signals were numbered from 1 to 14 and assigned to the appropriate hydrogen-atom-containing molecular group. The ^1^H NMR spectra of 105 °C-treated and EB-irradiated samples of ARP confirmed the lack of influence of the formulation on the ARP structure (Figure 1). Previously, the satisfactory flow of P(l-LA:GA:TMC) during HME was achieved with pointed conditions in studies on P(l-LA:GA:TMC) rods with 17-β-estradiol (E_2_) or risperidone [9,10]. The formulation temperature should not exceed the *T_m_* of the drug substance and should be higher than *T_g_* in the case of amorphous polymers. However, it should be noted that completely amorphous polymers do not exist.

**Figure 2:**
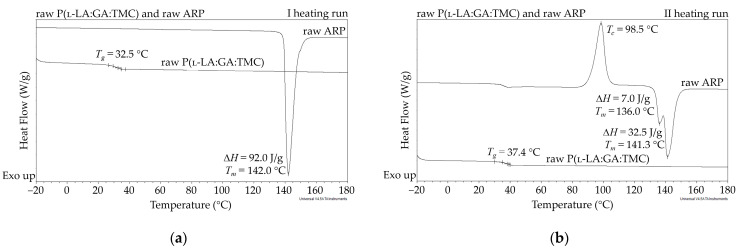
DSC curves for the first (**a**) and second (**b**) heating run of raw ARP and raw P(l-LA:GA:TMC). *T_c_*—crystallization temperature; *T_g_*—glass transition temperature; *T_m_*—melting temperature; Δ*H*—melting enthalpy.

In this study, the DSC analysis of ARP revealed a *T_m_* at 142.0 °C and *T_g_* of P(l-LA:GA:TMC) at 37.4 °C (Figure 2); therefore, 105.0 °C was found to be appropriate for rod formulation. Moreover, the cold crystallization of ARP at 98.5 °C and re-melting at 136.0 °C and 141.3 °C were noted, but were irrelevant to the quality of the drug substance (Figure 2). The comparison of the ^1^H NMR spectra of raw ARP, 105 °C-treated ARP, and EB-irradiated ARP showed no significant differences in the quality, quantity, or intensity of signals in the range of 10.0 to 1.5 ppm between the tested samples. The results clearly indicate the lack of influence of temperature on the ARP structure during HME and EB irradiation (Figure 1).

The most important aspect of polymer formulations is the drug loading ability. Generally, in the case of HME, a high efficiency is achieved, which was also confirmed in this study. ARP loading into the P(l-LA:GA:TMC) structure determined by ^1^H NMR spectroscopy was achieved at the level of 94.3 ± 1.4%.

**Figure 3:**
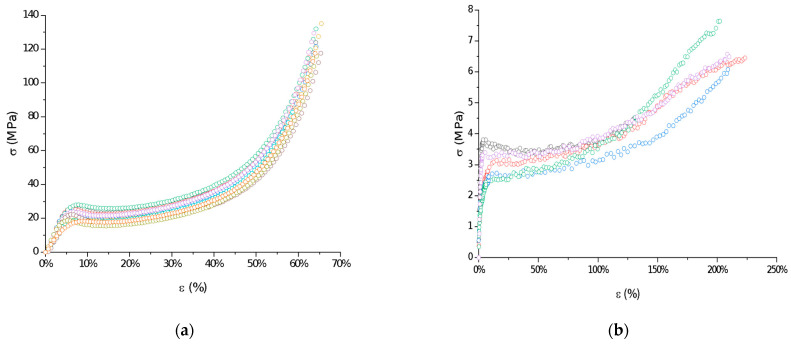
Examples of stress–strain curves of raw P(l-LA:GA:TMC): compression test (**a**) and tensile test (**b**).

The mechanical properties of raw P(l-LA:GA:TMC) under compression and tensile loading were determined. Stress–strain curves showed the ductile mode of deformation (Figure 3). Strain hardening was noted for all samples, probably because of polymer chain/network reorganization during deformations [18]. In this study, the Young’s modulus at compression and tension (386.21 ± 86.02 MPa and 105.97 ± 25.29 MPa, respectively) and both yield (22.57 ± 3.21 MPa and 3.10 ± 0.42 MPa, respectively) and breaking stresses (6.24 ± 1.18 MPa) were noted (Table 2). The obtained mechanical data had significantly lower values compared to the results of other studies on poly(l-lactide) or poly(l-lactide-*co*-glycolide) copolymers [16,19,20], which may reflect the influence of crystallinity on mechanical properties. Generally, lower parameters were noted for amorphous materials than for semicrystalline or crystalline materials. A lack of endothermal events for raw P(l-LA:GA:TMC) and native rods may suggest that they have a more amorphous character. Moreover, the reduction in the values for their mechanical properties may also result from the rubber-like behavior described for TMC, which may act as a plasticizer [21,22], resulting in the high level of deformation of the studied P(l-LA:GA:TMC) under lower mechanical stress. The plastification effect was also confirmed by the reduction of *T_g_* by the addition of TMC to terpolymer compared with PLGA copolymers [16,19,20].

The reduction in the indicated mechanical values is a significant advantage in the case of rods. It was noted that a low Young’s modulus, ductile deformations, and high breaking strain are crucial for minimally invasive delivery systems such as syringe-injectable hydrogels [23]. However, one of the major drawbacks of the use of hydrogels as drug formulations is that they cannot be removed if side effects occur.

**Figure 4:**
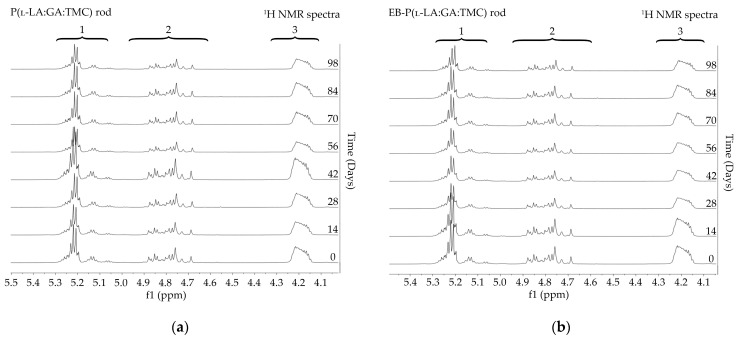
^1^H NMR spectra (600 MHz, DMSO-d6) of the P(l-LA:GA:TMC) rods (**a**) and the EB-P(l-LA:GA:TMC) rods (**b**) during degradation. ^1^H NMR spectra: methine proton region of the lactidyl units (1), and methylene proton region of the glycolidyl (2) and carbonate units (3).

**Figure 5:**
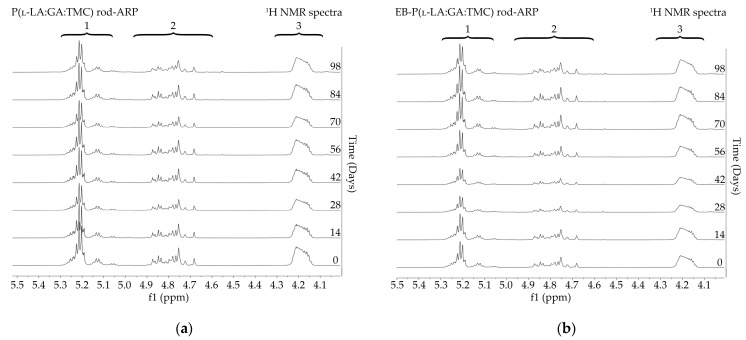
^1^H NMR spectra (600 MHz, DMSO-d6) of the P(l-LA:GA:TMC) rods-ARP (**a**) and the EB-P(l-LA:GA:TMC) rods-ARP (**b**) during degradation. ^1^H NMR spectra: methine proton region of the lactidyl units (1), and methylene proton region of the glycolidyl (2) and carbonate units (3).

**Figure 6:**
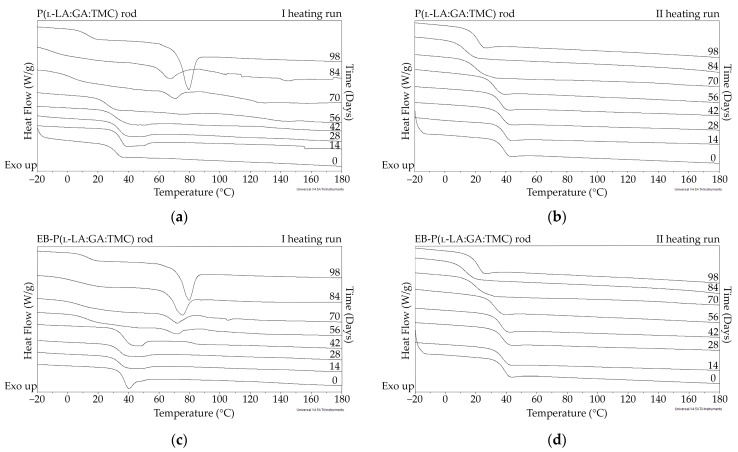
DSC curves of the first (**a**,**c**) and the second heating runs (**b**,**d**) of P(l-LA:GA:TMC) rods (**a**,**b**) and EB-P(l-LA:GA:TMC) rods (**c**,**d**).

**Figure 7:**
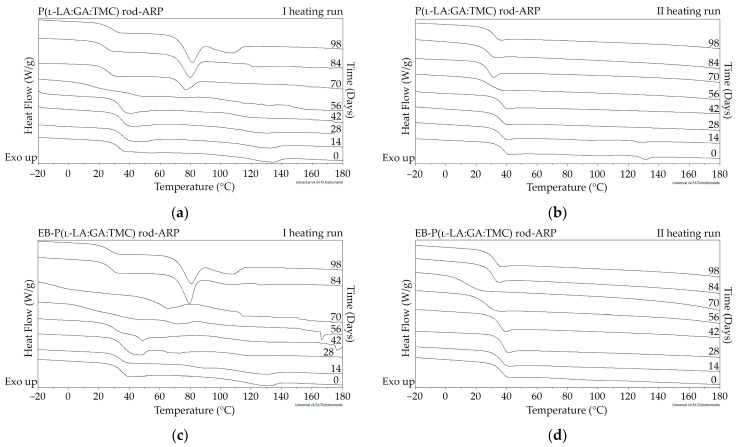
DSC curves of the first (**a**,**c**) and the second heating runs (**b**,**d**) of P(l-LA:GA:TMC) rods-ARP (**a**,**b**) and EB-P(l-LA:GA:TMC) rods-ARP (**c**,**d**).

**Figure 8:**
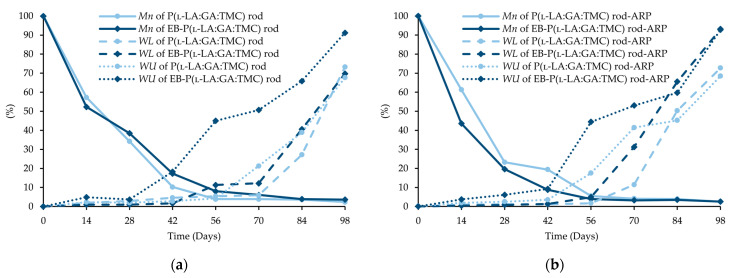
Changes (%) in molecular weight (*M_n_*), weight loss (*WL*), and water uptake (*WU*) during the degradation of the terpolymer rods: P(l-LA:GA:TMC) rods and EB-P(l-LA:GA:TMC) rods (**a**); P(l-LA:GA:TMC) rods-ARP and EB-P(l-LA:GA:TMC) rods-ARP (**b**).

The measurements of NMR, DSC, and GPC for P(l-LA:GA:TMC) rods, EB-P(l-LA:GA:TMC) rods, P(l-LA:GA:TMC) rods-ARP, and EB-P(l-LA:GA:TMC) rods-ARP (Figure 4, Figure 5, Figure 6, Figure 7 and Figure 8, Table 3 and Table 4) revealed only negligible changes in the analyzed parameters, except for the *T_g_* and *M_n_* resulting from HME and EB irradiation compared to raw terpolymer (Figure 2 and Table 1). The initial *T_g_* of raw P(l-LA:GA:TMC) was 37.4 °C (Figure 2 and Table 1), and the formulation of P(l-LA:GA:TMC) rods using HME did not affect this parameter, whereas EB irradiation slightly decreased the *T_g_* to 36.6 °C (Figure 6, Table 3). The loading of ARP into rods did not influence the *T_g_* additionally, whereas the loading of ARP into rods and EB irradiation resulted in a further decrease in the *T_g_* value to 35.6 °C (Figure 7 and Table 4). Another study revealed that EB irradiation may influence the decrease in *T_g_*, which is related to chain scission [13].

The intensity of the *M_n_* changes depends on the properties of the drug substance, the polymer material used, and the processing conditions during HME [10,24]. The decrease in *M_n_* as the result of extrusion may even be up to 64% [24]. However, it was pointed out that *M_n_* is only one of several variables characterizing drug delivery systems [9,25]. In this study, a decrease in *M_n_* as a result of HME from 77.1 kDa to 46.8 kDa for P(l-LA:GA:TMC) rods and to 43.9 kDa for P(l-LA:GA:TMC) rods-ARP (Table 1, Table 3 and Table 4) was observed. The further decrease may result from EB irradiation [13]. In this study, *M_n_* was 41.1 kDa for EB-P(l-LA:GA:TMC) rods, whereas for EB-P(l-LA:GA:TMC) rods-ARP it was 43.4 kDa as a result of the EB irradiation (Table 3 and Table 4).

Degradation revealed differences in the changes in structural and thermal parameters between rods. Generally, the changes in the terpolymer content were gradual, suggesting the stable and steady degradation of all rods. Only on the last days of degradation were greater changes seen (Figure 4 and Figure 5, Table 3 and Table 4).

**Figure 9:**
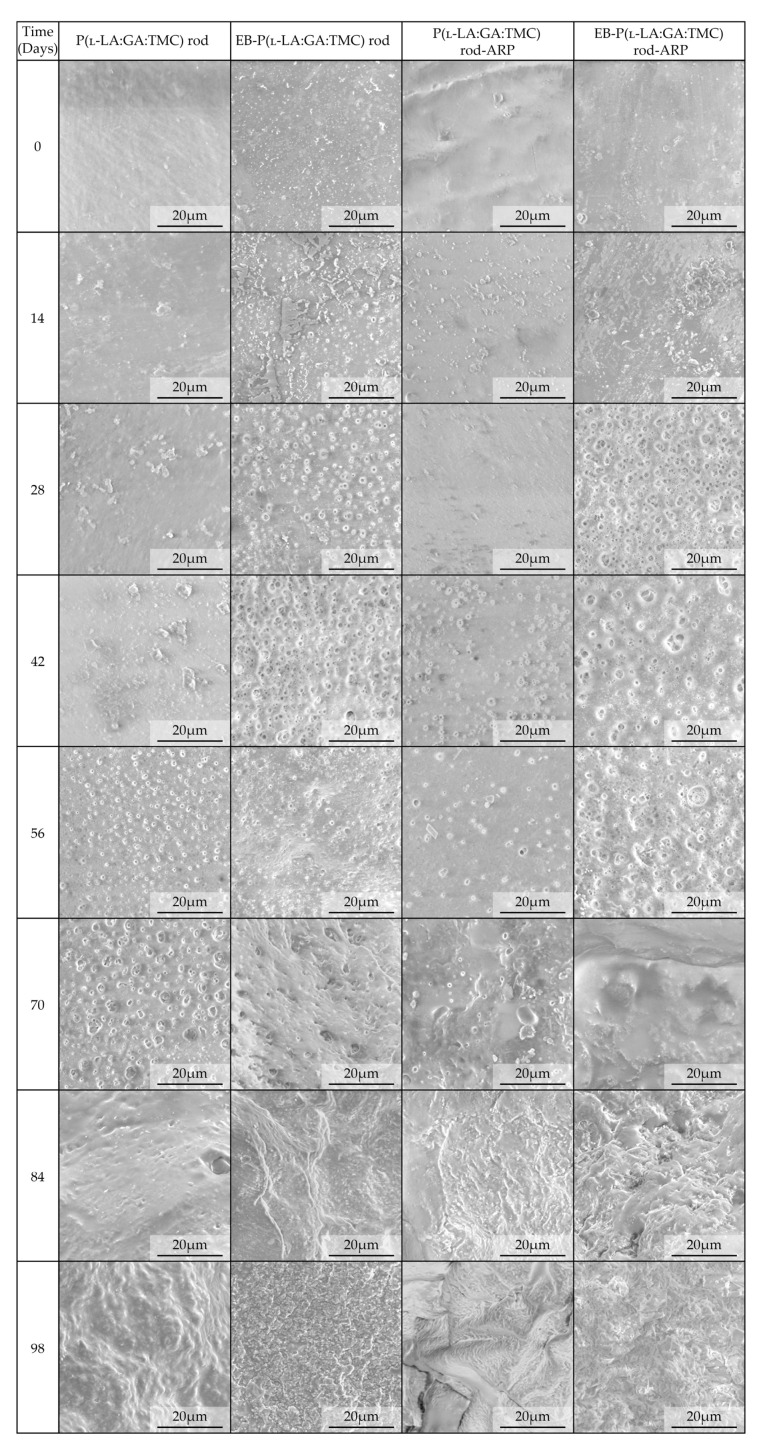
SEM images of terpolymer rods during degradation.

Generally, the changes in the structural and thermal parameters occurred as a result of the hydrolysis of ester bonds of P(l-LA:GA:TMC). Structural changes were intensified by an increase in *WU* (Figure 8, Table 3 and Table 4) and the formation of pores (Figure 9). It should be noted that the presence of drug substances also contributes to the changes in *WU*. Formulating rods using the HME method makes the structure particularly solid. However, the incorporation of drug substances into the polymer matrix resulted in the formation of a looser structure, which may be more susceptible to *WU*. Loo and coworkers’ study pointed out that the increase in *WU* may be due to the presence of more hydrophilic end groups as a result of the chain scission caused by EB irradiation [13].

**Figure 10:**
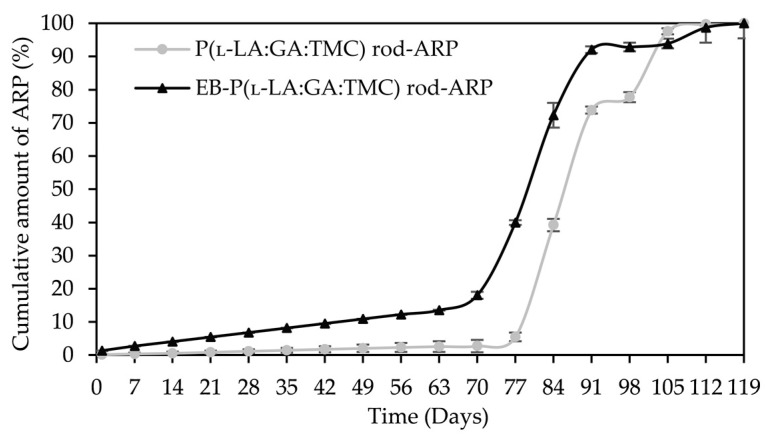
ARP cumulative release profiles.

The analysis of this parameter on the 56th day of the experiment can be considered representative, taking into account the release profiles (Figure 10). For P(l-LA:GA:TMC) rods, P(l-LA:GA:TMC) rods-ARP, EB-P(l-LA:GA:TMC) rods, and EB-P(l-LA:GA:TMC) rods-ARP, the increase in *WU* was 4.2%, 17.5%, 45.2%, and 44.4% by the 56th day, respectively (Table 3 and Table 4). Undoubtedly, the released drug substance created spaces that were filled with water, which may have also facilitated degradation. Moreover, the chain scission of P(l-LA:GA:TMC) caused by the EB irradiation accelerated the degradation changes, as mentioned above.

For EB-irradiated rods, the earliest pore formation was observed as a result of faster *WU* or vice versa. This effect reflected the study of Loo and co-workers, in which microcavities in EB-irradiated PLGA film facilitated degradation [13]. In the case of P(l-LA:GA:TMC) rods and P(l-LA:GA:TMC) rods-ARP, the pores were observed on the 56th and 42nd days, respectively, while for EB-P(l-LA:GA:TMC) rods and EB-P(l-LA:GA:TMC) rods-ARP they were observed on the 28th day of degradation (Figure 9). These phenomena may have influenced the *M_n_* and *WL* changes as a result of hydrolytic degradation (Table 3 and Table 4).

The thermal analysis of the first heating run revealed that during the early period of P(l-LA:GA:TMC) rods-ARP and EB-P(l-LA:GA:TMC) rods-ARP degradation (0–14 days), the visible endothermic event at 134.0 °C was due to the ARP, whereas for blank rods no additional events were observed, which may indicate that all terpolymer rods were amorphous at the beginning of incubation in PBS (Figure 2, Figure 6 and Figure 7). However, some crystalline fragments may have been present in the terpolymer structure. For the EB-P(l-LA:GA:TMC) rods on the 56th day of degradation, an endothermic event was observed, whereas for the rest of the rods it was observed on the 70th day of degradation. All these changes may indicate that a crystallization and/or ordering process took place in the terpolymer matrix (Figure 6 and Figure 7, Table 3 and Table 4).

The presence of ARP influenced the characteristics of the changes in *T_g_* during degradation. P(l-LA:GA:TMC) rods and EB-P(l-LA:GA:TMC) rods showed decreases from 37.3 °C to 19.9 °C and from 36.6 °C to 20.0 °C, respectively (Table 3), while for P(l-LA:GA:TMC) rods-ARP and EB-P(l-LA:GA:TMC) rods-ARP, the changes were smaller. The *T_g_* decreased from 37.4 °C to 30.7 °C and from 35.6 °C to 30.6 °C, respectively (Table 4). ARP may have acted as an anti-plasticizer during degradation because smaller changes in *T_g_* in comparison to those in the blank terpolymer rods were seen. For all rods, fluctuations in the *T_g_* values were observed, which may point to the ordering of the terpolymer. In the final days of degradation, the values of *T_g_* increased (Table 3 and Table 4). Analogous changes were observed in previous studies on the release of E_2_ from P(l-LA:GA:TMC) rods [9]. It should be noted that, due to the low content of drug substances in the final phase of the release process, the changes in *T_g_* do not play a significant role.

A decrease in *M_n_* was observed for all rods during degradation. In the case of EB-P(l-LA:GA:TMC)-ARP rods, the decrease in *M_n_* was faster compared to that in other rods (Figure 8).

In our opinion, an increase in *WL* is direct proof of the degradation process taking place. The changes in the other parameters provided information about the structural, morphological, and thermal modification or order of the structure. The fastest increase in *WL* was observed for EB-irradiated rods and may have resulted from chain scission (Figure 8, Table 3 and Table 4).

A tri-phasic model of ARP release was revealed for the analyzed rods. However, the differences in the degradation features of individual rods influenced the differences in the release pattern of ARP (Figure 10). From P(l-LA:GA:TMC) rods-ARP, the drug substance was released over 119 days in three phases: (i) the lag phase of release without a burst effect (77 days—5.0%), (ii) the main release phase with drug diffusion (28 days—92.0%), and (iii) the last release phase related to erosion (14 days—3.0%) (Figure 10). However, for EB-P(l-LA:GA:TMC) rods-ARP, differences in the release of ARP were observed as follows: (i) the duration of the lag phase was shorter (70 days), with a larger amount of released ARP (18.0%) as a result of faster *WU* (Table 4) and earlier pore formation (Figure 9); (ii) the main release phase was shorter (21 days), with a lower amount of ARP released (76.0%); (iii) the last release phase was longer (28 days), with a larger amount of ARP released (6.0%) (Figure 10). Generally, the first phase of release from the rods can be attributed to the liberation of small amounts of non-entrapped ARP in the matrix of the terpolymer or ARP remaining close to the formulation surface. The lack of ARP clusters on the surface (Figure 9) and the high efficiency of the loading of ARP into the P(l-LA:GA:TMC) structure by HME influenced a low ARP release in the first phase. Additionally, no unfavorable burst effect was observed, which might arise from the lack of slits and cracks on the surface of the rod (Figure 9). The faster *WU* and earlier pore formation observed for EB-P(l-LA:GA:TMC) rods-ARP resulted in a faster degradation process correlated with changes in the release pattern (Figure 10, Table 4). EB irradiation accelerated the release process. Additionally, for EB-P(l-LA:GA:TMC)-ARP rods, no deteriorations in the release profile were observed. Moreover, it can be pointed out that EB irradiation influenced a more stable degradation (Table 4). However, the changes in *M_n_*, *WU*, and *WL* were more dynamic, which may have favored the lack of deterioration in the release profile (Figure 10 and Table 4). A long lag phase for rods with ARP may be used in the design of a delayed release system in cases where one medicinal product is replaced with another.

## 4. Conclusions

The mechanical, structural, and thermal properties of P(l-LA:GA:TMC) are among the key features in the formulation of implantable and biodegradable rods with ARP. Compression tests indicated that rods may be safely administered with a pre-filled syringe due to the low stiffness and ductile mode of deformation under mechanical stresses. The analysis of the structural and thermal research allowed for the design of rods with ARP while preserving the ARP structure.

Moreover, degradation revealed the stable and steady characteristics of all rods. However, the differences in the changes in structural and thermal parameters were dependent on the presence of ARP and EB irradiation. In the case of rods with ARP, EB irradiation caused a chain scission of P(l-LA:GA:TMC), which intensified the degradation process. For EB-P(l-LA:GA:TMC) rods-ARP, a dynamic increase in pore formation allowed faster *WU*, favoring the hydrolytic degradation of ester bonds, which influenced a faster increase in *WL*. These effects accelerated the release of the drug substance from EB-P(l-LA:GA:TMC) rods-ARP compared to P(l-LA:GA:TMC) rods-ARP.

The proposed EB-P(l-LA:GA:TMC) rods-ARP may be used as a delayed release system in cases where one medicinal product needs to be replaced with another. The changes in the structural and thermal properties brought about by EB irradiation resulted in a gradual release of ARP.

## Figures and Tables

**Table 1:** Parameters used for characterizing raw P(l-LA:GA:TMC) for rod formulation.

Raw P(l-LA:GA:TMC)						
***F_LL_***	***F_GG_***	***F_TMC_***	***T_m_*** **(°C)**	***T_g_*** **(°C)**	***M_n_*** **(kDa)**	***D***
58.2	18.3	23.5	*ND*	37.4	77.1	2.001

*F_LL_*—molar percentage of lactidyl units in terpolymer; *F_GG_*—molar percentage of glycolidyl units in terpolymer; *F_TMC_*—molar percentage of carbonate units in terpolymer; *T_m_*—melting temperature; *T_g_*—glass transition temperature; *M_n_*—molecular weight; *D*—molecular weight distribution; *ND*—non-detected.

**Table 2:** Mechanical properties of raw P(l-LA:GA:TMC) under compression and tensile loading presented as means and standard deviations (±SD).

	ε_y_ (%)	σ_y_ (MPa)	ε_b_ (%)	σ_b_ (MPa)	E (MPa)
**Compression**	6.77 ± 1.01	22.57 ± 3.21	-	-	386.21 ± 86.02
**Tensile**	9.59 ± 4.84	3.10 ± 0.42	191.81 ± 35.33	6.24 ± 1.18	105.97 ± 25.29

ε_y_—yield strain; σ_y_—yield stress; ε_b_—breaking (identical to maximum) strain; σ_b_—breaking (identical to maximum) stress; E—Young’s modulus.

**Table 3:** Parameters characterizing the terpolymer rods during degradation.

**P(l-LA:GA:TMC) Rod**										
**Time (Days)**	***F_LL_***	***F_GG_***	***F_TMC_***	***T_m_*** **(°C)**	**∆*H*** **(J/g)**	***T_g_*** **(°C)**	***M_n_*** **(kDa)**	***D***	***WU*** **(%)**	***WL*** **(%)**
0	58.3	18.3	23.4	*ND*	*ND*	37.3	46.8	2.264	0	0
14	57.8	18.4	23.8	*ND*	*ND*	37.9	26.8	2.056	2.3	1.9
28	58.1	18.2	23.7	*ND*	*ND*	35.1	16.0	2.247	2.0	2.9
42	58.3	18.0	23.7	*ND*	*ND*	35.1	4.8	2.559	2.8	4.6
56	59.6	17.2	23.2	*ND*	*ND*	31.4	1.8	3.934	4.2	5.4
70	55.9	16.9	27.2	70.5	5.1	20.2	1.8	5.365	21.3	5.9
84	58.4	14.7	26.9	66.8	7.1	13.2	1.7	3.014	38.9	27.2
98	64.6	14.1	21.3	79.7	12.4	19.9	1.1	4.749	67.7	73.3
**EB-P(l-LA:GA:TMC) Rod**										
**Time (Days)**	***F_LL_***	***F_GA_***	***F_TMC_***	***T_m_*** **(°C)**	**∆*H*** **(J/g)**	***T_g_*** **(°C)**	***M_n_*** **(kDa)**	***D***	***WU*** **(%)**	***WL*** **(%)**
0	59.7	17.6	22.7	*ND*	*ND*	36.6	41.1	2.012	0	0
14	59.4	17.8	22.8	*ND*	*ND*	35.1	21.5	2.343	4.9	1.0
28	59.6	17.5	22.9	*ND*	*ND*	35.0	15.8	2.095	3.7	1.0
42	53.1	15.3	31.6	*ND*	*ND*	32.8	7.1	2.680	18.3	1.7
56	54.2	14.0	31.8	71.8	3.0	22.9	3.3	2.684	45.2	11.3
70	54.2	14.1	31.7	72.1	4.5	17.6	2.5	2.006	50.7	12.2
84	57.7	13.9	28.4	75.4	10.3	18.9	1.6	5.640	65.9	40.6
98	60.2	12.8	27.0	80.1	10.4	20.0	1.5	3.205	91.2	69.8

*F_LL_*—molar percentage of lactidyl units in terpolymer; *F_GG_*—molar percentage of glycolidyl units in terpolymer; *F_TMC_*—molar percentage of carbonate units in terpolymer; *T_m_*—melting temperature; Δ*H*—melting enthalpy; *T_g_*—glass transition temperature; *M_n_*—molecular weight; *D*—molecular weight distribution; *WU*—water uptake; *WL*—weight loss; *ND*—non-detected.

**Table 4:** Parameters characterizing the terpolymer rods with ARP during degradation.

**P(l-LA:GA:TMC) Rod-ARP**										
**Time (Days)**	***F_LL_***	***F_GA_***	***F_TMC_***	***T_m_*** **(°C)**	***∆H*** **(J/g)**	***T_g_*** **(°C)**	***M_n_*** **(kDa)**	***D***	***WU*** **(%)**	***WL*** **(%)**
0	58.3	18.2	23.5	ND	ND	37.4	43.9	1.928	0	0
14	58.1	18.2	23.7	ND	ND	35.3	26.9	2.234	1.9	1.0
28	58.1	17.9	24.0	ND	ND	36.6	10.2	3.132	2.5	1.1
42	58.1	17.7	24.2	ND	ND	36.1	8.5	2.671	3.5	1.1
56	57.2	16.5	26.3	ND	ND	28.3	2.4	3.957	17.5	1.7
70	53.4	14.9	31.7	76.3	4.4	26.7	1.8	5.386	41.4	11.4
84	54.0	12.9	33.1	79.3	8.0	26.7	1.7	3.955	45.2	50.3
98	61.5	12.2	26.3	80.2 108.1	10.1 4.4	30.7	1.1	2.228	68.5	72.7
**EB-P(l-LA:GA:TMC) Rod-ARP**										
**Time (Days)**	***F_LL_***	***F_GA_***	***F_TMC_***	***T_m_*** **(°C)**	***∆H*** **(J/g)**	***T_g_*** **(°C)**	***M_n_*** **(kDa)**	***D***	***WU*** **(%)**	***WL*** **(%)**
0	59.4	17.5	23.1	ND	ND	35.6	43.4	2.012	0	0
14	59.6	16.7	23.7	ND	ND	35.1	18.9	2.086	3.7	0.4
28	59.6	16.6	23.8	ND	ND	36.4	8.5	2.401	6.1	0.8
42	49.4	16.1	34.5	ND	ND	33.7	3.8	2.559	9.2	1.3
56	48.5	14.6	36.9	ND	ND	26.0	1.7	3.646	44.4	4.9
70	57.8	15.0	27.2	75.0	4.1	26.6	1.4	4.579	53.0	31.2
84	47.0	20.4	32.6	79.6	9.4	29.4	1.5	1.858	59.7	65.5
98	62.3	14.2	23.5	80.1 106.2	10.5 4.0	30.6	1.1	2.301	92.8	93.1

*F_LL_*—molar percentage of lactidyl units in terpolymer; *F_GG_*—molar percentage of glycolidyl units in terpolymer; *F_TMC_*—molar percentage of carbonate units in terpolymer; *T_m_*—melting temperature; Δ*H*—melting enthalpy; *T_g_*—glass transition temperature; *M_n_*—molecular weight; *D*—molecular weight distribution; *WU*—water uptake; *WL*—weight loss; *ND*—non-detected.

## Data Availability

The data presented in this study are available on request from the corresponding author.
